# PhenoDis: a comprehensive database for phenotypic characterization of rare cardiac diseases

**DOI:** 10.1186/s13023-018-0765-y

**Published:** 2018-01-25

**Authors:** Angela Adler, Pia Kirchmeier, Julian Reinhard, Barbara Brauner, Irmtraud Dunger, Gisela Fobo, Goar Frishman, Corinna Montrone, H.-Werner Mewes, Matthias Arnold, Andreas Ruepp

**Affiliations:** 1 Institute for Bioinformatics and Systems Biology (IBIS), Helmholtz Zentrum München, German Research Center for Environmental Health (GmbH), D-85764 Neuherberg, Germany; 2 Technische Universität München, Chair of Genome Oriented Bioinformatics, Center of Life and Food Science, D-85350 Freising-Weihenstephan, Germany

**Keywords:** Rare cardiac diseases, Heart, Phenotype, Genotype, Precision medicine, Genetic disorders, Decision support systems, Bioinformatics, Medical genetics, Personalized medicine

## Abstract

**Background:**

Thoroughly annotated data resources are a key requirement in phenotype dependent analysis and diagnosis of diseases in the area of precision medicine. Recent work has shown that curation and systematic annotation of human phenome data can significantly improve the quality and selectivity for the interpretation of inherited diseases. We have therefore developed PhenoDis, a comprehensive, manually annotated database providing symptomatic, genetic and imprinting information about rare cardiac diseases.

**Results:**

PhenoDis includes 214 rare cardiac diseases from Orphanet and 94 more from OMIM. For phenotypic characterization of the diseases, we performed manual annotation of diseases with articles from the biomedical literature. Detailed description of disease symptoms required the use of 2247 different terms from the Human Phenotype Ontology (HPO). Diseases listed in PhenoDis frequently cover a broad spectrum of symptoms with 28% from the branch of ‘cardiovascular abnormality’ and others from areas such as neurological (11.5%) and metabolism (6%). We collected extensive information on the frequency of symptoms in respective diseases as well as on disease-associated genes and imprinting data. The analysis of the abundance of symptoms in patient studies revealed that most of the annotated symptoms (71%) are found in less than half of the patients of a particular disease. Comprehensive and systematic characterization of symptoms including their frequency is a pivotal prerequisite for computer based prediction of diseases and disease causing genetic variants. To this end, PhenoDis provides in-depth annotation for a complete group of rare diseases, including information on pathogenic and likely pathogenic genetic variants for 206 diseases as listed in ClinVar. We integrated all results in an online database (http://mips.helmholtz-muenchen.de/phenodis/) with multiple search options and provide the complete dataset for download.

**Conclusion:**

PhenoDis provides a comprehensive set of manually annotated rare cardiac diseases that enables computational approaches for disease prediction via decision support systems and phenotype-driven strategies for the identification of disease causing genes.

## Background

Cardiovascular disease (CVD), in its manifold appearances, is one of the leading causes of mortality and morbidity in the Western world. The 2013 Global Burden of Disease study estimated that CVD caused 17.3 million deaths globally, accounting for 31.5% of all deaths and 45% of all non-communicable disease deaths [1]. CVD covers a wide spectrum of highly diverse diseases ranging from highly prevalent complex diseases (coronary heart disease, stroke) to a multitude of rare cardiac diseases that frequently include a strong genetic component. For rare cardiac diseases, low prevalence or overlapping manifestations are major obstacles for correct diagnosis. Precise phenotyping followed by integrated computational analysis of genotype and phenotype is thus becoming increasingly important for both diagnostics and translational research. The growing availability of digitalized medical information in electronic health records and the application of portable devices for medical monitoring will further promote the development and application of computational analysis tools [2, 3].

With the manifold possible manifestations of CVD, careful characterization of the phenotype is of particular importance. The ability to differentiate between diseases on the basis of symptom information relies on specialized databases providing comprehensive and standardized specifications of the individual diseases. To be accessible by computational means, these specifications need to be structured and organized following the principles of a standardized ontology, i.e. a structured representation of interrelated terms. The two largest public resources addressing this need are OMIM (Online Mendelian Inheritance in Man) and Orphanet. OMIM is a comprehensive, authoritative compendium of human genes and genetic phenotypes, containing information on all known Mendelian disorders [4]. The information on symptoms belonging to a disease is primarily presented as free text but is also available as a list of short terms similar to a biomedical vocabulary. Orphanet is a European initiative with multiple information resources from the field of rare diseases that are curated by health experts [5]. Yet, Orphanet maintains an ontology to provide a structured vocabulary for rare diseases. While the set of diseases listed in OMIM and Orphanet overlap, the highly valuable content of the two resources differs significantly in composition, such as granularity in disease terminologies, specificity of symptom descriptions, and the vocabulary of specifications, limiting their utility to thoroughly annotate particularly rare CVDs. Future progress in the diagnosis and investigation of CVDs, in particular the support through computer-based methods requires a comprehensive, systematic and homogeneous annotation of individual diseases. With the advent of next-generation sequencing (NGS)-based diagnostics in the clinic, symptom-based approaches are increasingly complemented by targeted screening for pathogenic genetic variants using gene panel-based sequencing platforms. As the genotype of known causal genetic variants provides the strongest discriminator between rare or monogenic diseases, NGS is a highly valuable addition to the diagnostic toolbox. Modern resources for disease annotation are therefore required to include both a comprehensive specification of symptom-based medicine and the current catalog of known causal genetic variants.

## Methods

Here we present PhenoDis, a comprehensive database for rare cardiac diseases that provides an interface to disorders listed in both resources in a structured way, augmented by additional information from biomedical literature. PhenoDis offers detailed symptomatic annotation for 308 rare cardiac diseases using the Human Phenotype Ontology, the state-of-the-art vocabulary for phenotypic prediction of diseases. Stratification of particular diseases is further supported by frequency data of symptoms in respective diseases, presentation of disease-linked genetic variants and epidemiological information. We demonstrate the value of PhenoDis by comparing it to OMIM and Orphanet. We propose and elaborate future applications of PhenoDis for diagnostic approaches.

## Results and discussion

### Construction and content

#### The PhenoDis dataset of rare cardiac diseases

Due to the high number of existing nomenclatures and classifications of diseases with own structures and vocabularies, we compiled a set of specific descriptors dedicated for characterization of rare cardiac diseases. We made use of information from two highly accepted resources for disease information, Orphanet and OMIM.

Orphanet is a comprehensive European resource with information on rare diseases [5]. Among the 33 groups of rare diseases that were grouped and classified by Orphanet is the category ‘rare cardiac diseases’. This category consists of 214 different diseases based on the release of September 2016. The Orphanet classification is organized in a hierarchical structure where specific diseases such as ‘Carney complex’ and ‘Familial atrial myxoma’ are subgroups of higher level terms like ‘Rare cardiac tumor’. As symptoms of more general disease groups are rather unspecific, we usually did not annotate these diseases but referred to annotations on a more specific level. Only exceptions are diseases such as Brugada syndrome where disease subtypes cannot be distinguished on the symptom level. In total, we referred in 65 cases to annotations of diseases on a more fine-grained level. Our dataset includes diseases that are obviously specific to the heart such as cardiomyopathies but also diseases such as alpha-mannosidosis with only weak cardiac symptomatic manifestation. It can be assumed that this dataset will change over time as organizations revise their datasets. Beta-mannosidosis (ORPHA118) and Kawasaki disease (ORPHA2331) for example were formerly assigned to rare cardiac diseases but have been reclassified recently.

The great majority of human Mendelian disorders have been described in detail in the OMIM database. Differences in the specificity of disease description between OMIM and Orphanet prompted us to use disease names from both resources. ‘Systemic sclerosis’ for example is covered in OMIM by one entry (SCLERODERMA, FAMILIAL PROGRESSIVE; %181,750) but is represented as ‘Systemic sclerosis’ and four subdiseases in Orphanet (Diffuse cutaneous systemic sclerosis; Limited cutaneous systemic sclerosis; Limited systemic sclerosis and CREST syndrome). On the other hand, OMIM displays a finer granularity than Orphanet e.g. in ‘Familial atrial fibrillation’. For PhenoDis, we included additional 94 rare cardiac diseases from OMIM [4], 44 of them subtypes of cardiomyopathies. It should be noted that the dataset of rare diseases in this first version of PhenoDis is focused on heart diseases concerning abnormalities in heart physiology and not on malformations of the cardiac system. We plan to extend the scope of PhenoDis to more groups of rare diseases in further versions.

#### Data acquisition by manual curation

The content of PhenoDis was compiled by critical reading of 1191 scientific articles as of March 2017. The PhenoDis biocurators have a long-standing track record in the annotation of biomedical databases [6, 7] and the interpretation of clinical results, e.g. in cardiomyopathies [8]. A key-challenge during generation of a database like PhenoDis is transforming information from cohort studies, case studies and reviews, which is mostly presented in natural language, into a structured and controlled vocabulary such as HPO (see below). To achieve this, information about disease symptoms, associated information and epidemiological findings such as prevalence, inheritance and age of onset were manually annotated. PubMed identifiers of all considered articles are also added, which allows users to retrieve more detailed information. This is especially useful for numbers reported on disease symptom frequency which to some extend differs between patient studies for various reasons. One example for this is the symptom ‘Telangiectasia of the skin’ in systemic sclerosis which was found in 39% (268 patients; [9]) and 18–24% (43 patients; [10]) of the patients in two studies, respectively. This could be due to ethnical differences, as the former study was conducted with patients from Europe and the US whereas the latter included patients from Japan.

#### HPO as global vocabulary

Clinical diagnostics is challenging in the field of medical genetics, as the differential diagnosis is often complicated by the large number of genetic disorders, each of which can be characterized by numerous clinical features that are often shared across many diseases. OMIM is the largest available collection of disease phenotypes but does not use a controlled vocabulary. This causes difficulties to recognize synonyms by computational means. For description of clinical symptoms various vocabularies like Human Phenotype Ontology (HPO), MedDRA, Orphanet Rare Disease ontology (ORDO) and SnoMed exist. The HPO was developed to provide a standardized vocabulary of phenotypic abnormalities encountered in human disease [11]. Similarly to the Gene Ontology [12], HPO is constructed as a directed acyclic graph allowing a term to have multiple parent terms. For example, ‘Calcific mitral stenosis’ (HP:0200129) is a ‘Mitral valve calcification’ (HP:0004382) as well as a ‘Mitral stenosis’ (HP:0001718). Analysis of simulated data has shown that the HPO is able to describe phenotypic similarity at different levels of granularity and that the calculation of similarity is not overly sensitive to noise, completeness, or specificity of the set of phenotypic terms [13]. The broad acceptance of HPO as symptom vocabulary is documented by its use in projects such as DDD [14], the UK 100,000 Genomes Project [14] and cross-references with terms from the OMIM clinical synopsis. In PhenoDis, we therefore manually converted free text symptom descriptions from the literature to the best-matching symptom term in HPO.

#### Symptom annotation

Information about rare cardiac diseases was obtained from patient studies, dedicated reviews and research articles from the biomedical literature. Depending on the number of available cases and scientific coverage of respective diseases, sources range from single case studies up to cohort studies with more than 1000 participants (e.g. in systemic sclerosis). If more than one study was available for a disease, we selected articles based on the number of patients, topicality and number of covered symptoms. PhenoDis contains a total number of 7582 disease-symptom relations, which means that one disease is on average related to 31.2 annotated symptoms. The term ‘cardiovascular abnormality’ is the root that covers all HPO terms that point to an abnormal phenotype in the cardiovascular system. From all annotated disease-symptom relations 72% do not belong to the ‘cardiovascular abnormality’ branch of HPO. This is a considerable fraction but it is not unexpected, as diseases such as ‘Glycogen storage disease due to acid maltase deficiency, infantile onset’ only show four cardiovascular symptoms and 21 others. If appropriate, symptom annotations are accompanied by a comment providing further specifics such as age-related changes. In ‘Carnitine uptake deficiency’ for example the symptom ‘hepatic encephalopathy’ appears at an infantile age, whereas ‘Easy fatigability’ is usually not seen before adulthood [15].

#### Symptom frequency

**Fig. 1:**
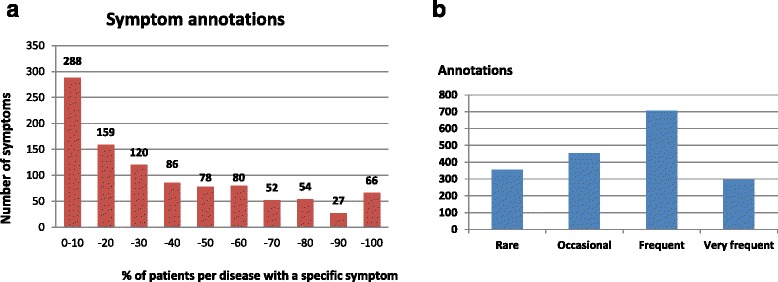
Distribution of symptom frequency and abundance in rare cardiac diseases. **a** Symptom abundance across all diseases displayed as percentage of patients per disease showing a specific symptom (grouped in 10% bins). For instance, 120 annotated symptoms were found in 20–30% of patients affected by a disease. **b** Frequency of symptom occurrence listed in databases and textual information from the literature, mapped to one of the four categories ‘Rare’ (< 7.5%), ‘Occasional’ (7.5%–32%), ‘Frequent’ (33–89%) and ‘Very frequent’ (≥90%)

Most symptoms of a disease are not present in all patients but only in a fraction. So far, this feature is extensively annotated only in the Orphant dataset [5]. For disease prediction this is a crucial information as it was shown that emphasizing infrequent features almost completely reduces recognizable biological coherence of phenotype clusters [16]. Therefore, we considered the inclusion of feature frequencies to be a requirement for optimal phenotype representation [16]. Consequently, we included the frequency of symptoms in PhenoDis annotations whenever this information was available. To achieve this, we used two types of information, frequency and abundance. We used frequency when exact numbers from patient studies were available. In total, we found 1000 symptom-frequency relations. In addition to the percentage of patients with a symptom we provide the number of participants in the related study. The distribution of frequencies (Fig. 1a) shows that the phenotype of diseases is dominated by symptoms that appear in a minority of patients. Only 9% of symptoms were found in more than 80% of patients with a particular disease whereas 40% of symptoms with known frequency are present in less than 20% of respective patients.

In addition, we give the distribution of symptom occurrences in four abundance categories: Very frequent (≥90%), Frequent (33–89%), Occasional (7.5%–32%) and Rare (< 7.5%). This allowed complementing the 1000 cases above with 808 abundancies that were retrieved from non-specific statements in scientific articles. The majority of symptoms are classified as Frequent (Fig. 1b). When comparing the distributions of symptom frequency and symptom abundance, one can immediately recognize that there is a striking difference between the small amount of frequencies between 90% and 100% and the abundance of Very frequent which represents 16.4% of all abundancies. This is presumably due to the usage of verbal expressions of symptom abundancies which are inconsistently or ambiguously used across different publications.

#### Epidemiological data

Whenever available in the literature we added epidemiological information to the dataset. The limit for prevalence was set to 1 patient in 2000 persons according to the definition of rare diseases by the European Commission on Public Health. We found prevalence data for 122 (40%) rare cardiac diseases with patient numbers as low as one case in Encephalopathy-hypertrophic cardiomyopathy-renal tubular disease. Information about the age of onset was found for 149 (48%) diseases. In contrast to metabolic diseases which manifest mostly early in life, the age of onset in rare cardiac diseases is found to be distributed from neonatal to adulthood. Published information about inheritance was found for 205 (67%) rare cardiac diseases in our current dataset. However, there are some diseases where the inheritance pattern is more complex and cannot be defined by classical terms such as autosomal dominant or autosomal recessive. An example is uniparental imprinting, where, via epigenetic regulation, only the paternal or maternal allele is expressed. In such cases a mutation in the expressed allele causes the disease, whereas the identical mutation in the allele that is silenced has no effect (Beckwith-Wiedemann syndrome due to imprinting defect of 11p15) [17].

#### Genetic data

In order to incorporate information on genetic variants and genes that are likely to be predisposing or causative for rare cardiac diseases, we used a customized version of the SNiPA variant annotation pipeline [18] with an updated version of the ClinVar database (April 29th, 2016) [19] to obtain the set of genetic variants that have been annotated as pathogenic or likely pathogenic in the context of rare cardiac diseases as well as their mapping to genes. 46,258 of 138,866 initial entries in ClinVar data were assigned as (likely) pathogenic, corresponding to 5030 distinct disease names. Of these, 284 disease names and 3266 genetic variants located in 206 distinct genes, respectively, could be mapped to 206 distinct diseases contained in PhenoDis. For 161 (78.2%) of the 206 diseases, variants annotated to be (likely) pathogenic were located in one single gene, while for the remaining 45 diseases variant data linked between 2 to 32 (mean = 2.54) genes, with one to 848 (mean = 30.65) variants mapped to each disease.

### Utility and discussion

#### User Interface

**Fig. 2:**
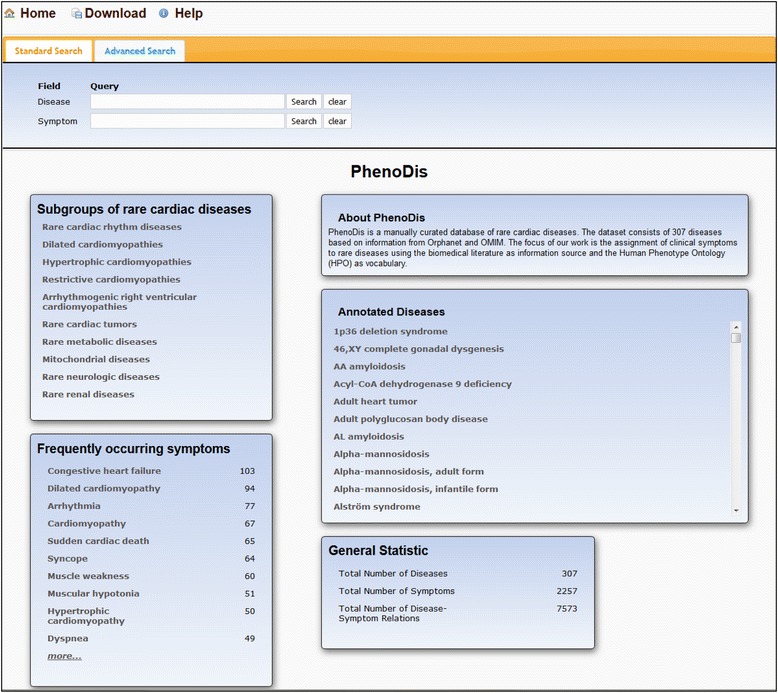
The PhenoDis homepage. Direct access to particular subsets of diseases is enabled via ‘Subgroups of rare cardiac diseases’ and ‘Frequently occurring symptoms’. The scrollbar on the right allows direct access to the alphabetically ordered disease list of PhenoDis

The PhenoDis dataset is accessible via a user-friendly interface (Fig. 2). In addition to disease names and symptoms that can be searched in the Standard search, the Advanced search allows including additional information such as epidemiological data or diseases within a specific disease classification. It also enables users to perform complex searches by using the Refine query option and the Boolean operators AND, OR and NOT. Complementary to the search options, all diseases of PhenoDis can be directly accessed via a scrollable list on the home page. The most frequently used symptoms in PhenoDis are shown on the home page and are linked to lists with the respective diseases. Annotation procedures and additional information can be found in the Help section. The dataset of all diseases can be downloaded as Tab-separated file.

#### Comparison of PhenoDis with OMIM and Orphanet

The two largest publicly available databases with information about rare diseases are OMIM and Orphanet [4, 5]. OMIM covers almost 5000 Mendelian phenotypes with known molecular basis. However, as OMIM does not provide a disease classification, it is not possible to filter for specific disease types such as rare cardiac diseases. Hence, generation of such a dataset requires a considerable manual effort. Phenotypic disease information in OMIM is predominately generated as free text in a semi-structured fashion and is accompanied with literature references. This kind of presentation is valuable for users who browse through the annotation of specific diseases but evades processing of the information by bioinformatics approaches. Hence, for 4000 diseases OMIM offers a clinical synopsis which resembles a vocabulary. However, the terms of the clinical synopsis are not controlled and thus can hardly be used by computational procedures in their current presentation. In PhenoDis, terms from the clinical synopsis are accompanied by literature references or by information about the symptom frequency. An analysis about the completeness of disease information resources has shown that for disease prediction purposes the content of the clinical synopsis gains significantly from additional symptoms from external resources [16]. Comparison of 51 annotated rare cardiac diseases in OMIM and PhenoDis reveals that PhenoDis provides on average 14.7 symptoms and OMIM 7.3 symptoms per disease. Such a quantitative comparison is an indication that symptom information in PhenoDis is either more granular with regard to the specificity of the symptoms or more comprehensive.

Orphanet is a resource with a focus on rare diseases [5]. Currently, the Orphanet disease classification assigns 214 diseases as rare cardiac. Recently, Orphanet changed symptom annotation from the Orphanet ontology (ORDO) to HPO. As of September 2016, Orphanet presents symptom annotations for 55 of the rare cardiac diseases. Compared to Orphanet, PhenoDis being the most comprehensive collection of rare cardiac diseases to our knowledge offers 255 symptom annotations. For 55 diseases that are characterized by both resources, Orphanet assigns 29 symptoms on average and PhenoDis 62. This could be due to the mapping from the smaller Orphanet Thesaurus (~ 1300 terms) to HPO (~ 11.877 terms). In contrast to PhenoDis, all symptoms in Orphanet were so far accompanied by one of three frequency terms (occasional, frequent, very frequent) given by the experts who characterized the respective diseases. The advantage of this approach is that in bioinformatics procedures every symptom has an applicable attribute. On the other hand, due to missing literature references, the validity of these frequencies cannot be evaluated which can be problematic in some cases. For instance, in ‘Systemic sclerosis’ the symptoms ‘pericarditis’ and ‘myositis’ are classified as ‘frequent’ in Orphanet (September 2016), although in a recent cohort study with 1037 Spanish patients these symptoms appeared only in 5% and 7% of cases, respectively (PMID: 24646463).

Overall, PhenoDis covers symptom annotation for 128 diseases that are not characterized by Orphanet (107) or OMIM (21).

#### Application of PhenoDis

To enable access to the comprehensive information on cardiac diseases contained in PhenoDis, we implemented a user-friendly interface that facilitates searches for diseases based on disease names or for diseases with specific symptoms (Fig. 2). An ‘Advanced search’ allows the selection of specific groups of diseases based on further attributes such as ‘age of onset’ or ‘disease classification’. The front page also offers links to subgroups of rare cardiac diseases such as mitochondrial diseases or rare neurological diseases. In addition, an extendable list of the most frequently annotated symptoms is available that links to lists of the respective diseases.

Although PhenoDis in its current form is not a disease prediction tool, the database search allows narrowing down the number of potential diseases for a given set of symptoms. For example, in 2013 Sabater-Molina and colleagues reported the case of a 30-year-old male with undiagnosed Barth syndrome [20]. Correct diagnosis was only suspected after recording of family history, thorough cardiologic, hematologic and metabolic screening. Yet, already at the age of 10 months, the patient was diagnosed with cardiac abnormalities, myopathy and showed recurrent infections. When querying for the latter two symptoms in the advanced search (“myopathy” AND “recurrent infections in infancy and early childhood”), PhenoDis would have suspected Barth Syndrome as only potential underlying disorder – solely based on the reported symptom composition.

Besides provision of the collected data, PhenoDis will enable the development of decision support systems, using the standardized vocabulary by HPO. A major obstacle in the correct clinical diagnosis is the large number of candidates resulting from the frequently overlapping occurrence of symptoms across rare diseases, leading to a high rate of delayed or even false diagnoses. A survey revealed that almost half (46%) of patients with rare diseases had to wait over 1 year for a final diagnosis following the onset of disease symptoms. One in five even had to wait over 5 years (https://www.raredisease.org.uk/media/1594/rduk-family-report.pdf). Computational approaches such as the Phenomizer show great promise to aid differential diagnosis. Prerequisite for these approaches is a comprehensive, coherently annotated dataset of diseases and disease symptoms. To the best of our knowledge PhenoDis is the first database describing a complete group of diseases with manual annotation that simultaneously use the Human Phenotype Ontology as vocabulary.

Along this line, applications for PhenoDis are envisioned in phenotype-driven strategies for the prioritization of potentially pathogenic variants in human genetic diseases. Large-scale clinical WES (whole exome sequencing) studies have reported a successful molecular diagnosis in up to 25% of cases in large cohorts of unselected, consecutive patients [21]. Despite this progress, the search for causal mutations can be difficult, if not guided by phenotypic information enabling to prioritize or discard loss-of-function mutations. Recent analyses have shown that computational phenotype analysis can substantially improve the performance of exome analysis pipelines. Using a strategy that combines information from exome sequencing and HPO-based phenotyping enabled diagnosis of acute ill patients within 26 h [22]. The study used a proprietary software for the analysis pipeline but there are also a number of freely available computational tools [14]. Detailed descriptions of clinical manifestations will thus be essential in future efforts to translate genetic findings for precision medicine. Together with genome information and data from other omics-methods, accurate and homogeneous phenotypic descriptions of rare diseases and disease subtypes will be a central component in the future diagnosis of diseases in both, science and the health care systems.

#### Future development

A major future improvement will involve the integration of novel studies from the literature. For diseases where currently only few information is available, inclusion of additional patient data will help to assign novel disease symptoms and to define statistical values, such as symptom frequency, more precisely. We plan to provide annual releases of PhenoDis including updates of database information such as changes in disease names and novel data from literature, e.g. from cohort studies. In addition, we plan to feed the PhenoDis disease information into data analysis tools. Prediction of rare cardiac diseases by submitting patient symptoms will be facilitated by an algorithm based on Phenomizer [23]. In a next step this tool will be extended to allow for analysing phenotypic and genotypic data in order to identify disease causing genes and gene variants.

## Conclusions

Precision medicine requires a detailed phenotypic description of diseases and the stratification of diseases into subtypes according to their underlying molecular mechanisms. PhenoDis provides a comprehensive characterization of 307 rare cardiac diseases with an emphasis on the phenotypic description. Prediction of diseases and disease-causing genes is facilitated by using the Human Phenotype Ontology (HPO) which is applied by the vast majority of computational approaches in the field. Characterization of the diseases also includes thoroughly manually curated epidemiological information and disease-associated genetic variants. A web interface with multiple search options as well, as the download of the complete dataset, is available at http://mips.helmholtz-muenchen.de/phenodis/.

## Abbreviations

CVD: Cardiovascular disease
DDD: Deciphering Developmental Disorders project
HPO: Human Phenotype Ontology
NGS: Next-generation sequencing
OMIM: Online Mendelian Inheritance in Man (web resource)

## References

[1] Townsend N, Wilson L, Bhatnagar P, Wickramasinghe K, Rayner M, Nichols M (2016). Cardiovascular disease in Europe: epidemiological update 2016. Eur Heart J.

[2] Jensen PB, Jensen LJ, Brunak S (2012). Mining electronic health records: towards better research applications and clinical care. Nat Rev Genet.

[3] Piwek L, Ellis DA, Andrews S, Joinson A (2016). The rise of consumer health Wearables: promises and barriers. PLoS Med.

[4] Amberger JS, Bocchini CA, Schiettecatte F, Scott AF, Hamosh A (2015). OMIM.org: online Mendelian inheritance in man (OMIM(R)), an online catalog of human genes and genetic disorders. Nucleic Acids Res.

[5] Rath A, Olry A, Dhombres F, Brandt MM, Urbero B, Ayme S (2012). Representation of rare diseases in health information systems: the Orphanet approach to serve a wide range of end users. Hum Mutat.

[6] Lechner M, Hohn V, Brauner B, Dunger I, Fobo G, Frishman G, Montrone C, Kastenmuller G, Waegele B, Ruepp A (2012). CIDeR: multifactorial interaction networks in human diseases. Genome Biol.

[7] Ruepp A, Waegele B, Lechner M, Brauner B, Dunger-Kaltenbach I, Fobo G, Frishman G, Montrone C, Mewes HW (2010). CORUM: the comprehensive resource of mammalian protein complexes--2009. Nucleic Acids Res.

[8] Kostareva A, Kiselev A, Gudkova A, Frishman G, Ruepp A, Frishman D, Smolina N, Tarnovskaya S, Nilsson D, Zlotina A (2016). Genetic Spectrum of idiopathic restrictive Cardiomyopathy uncovered by next-generation sequencing. PLoS One.

[9] van den Hoogen F, Khanna D, Fransen J, Johnson SR, Baron M, Tyndall A, Matucci-Cerinic M, Naden RP, Medsger TA, Carreira PE (2013). 2013 classification criteria for systemic sclerosis: an American College of Rheumatology/European league against rheumatism collaborative initiative. Arthritis Rheum.

[10] Makino K, Jinnin M, Makino T, Kajihara I, Fukushima S, Inoue Y, Ihn H (2014). Serum levels of soluble carbonic anhydrase IX are decreased in patients with diffuse cutaneous systemic sclerosis compared to those with limited cutaneous systemic sclerosis. Biosci Trends.

[11] Kohler S, Doelken SC, Mungall CJ, Bauer S, Firth HV, Bailleul-Forestier I, Black GC, Brown DL, Brudno M, Campbell J (2014). The human phenotype ontology project: linking molecular biology and disease through phenotype data. Nucleic Acids Res.

[12] Ashburner M, Ball CA, Blake JA, Botstein D, Butler H, Cherry JM, Davis AP, Dolinski K, Dwight SS, Eppig JT (2000). Gene ontology: tool for the unification of biology. The gene ontology consortium. Nat Genet.

[13] Robinson PN, Kohler S, Bauer S, Seelow D, Horn D, Mundlos S (2008). The human phenotype ontology: a tool for annotating and analyzing human hereditary disease. Am J Hum Genet.

[14] Smedley D, Robinson PN (2015). Phenotype-driven strategies for exome prioritization of human Mendelian disease genes. Genome Med.

[15] Magoulas PL, El-Hattab AW (2012). Systemic primary carnitine deficiency: an overview of clinical manifestations, diagnosis, and management. Orphanet J Rare Dis.

[16] Oti M, Huynen MA, Brunner HG (2009). The biological coherence of human phenome databases. Am J Hum Genet.

[17] Cerrato F, Sparago A, Verde G, De Crescenzo A, Citro V, Cubellis MV, Rinaldi MM, Boccuto L, Neri G, Magnani C (2008). Different mechanisms cause imprinting defects at the IGF2/H19 locus in Beckwith-Wiedemann syndrome and Wilms’ tumour. Hum Mol Genet.

[18] Arnold M, Raffler J, Pfeufer A, Suhre K, Kastenmuller G (2015). SNiPA: an interactive, genetic variant-centered annotation browser. Bioinformatics.

[19] Landrum MJ, Lee JM, Benson M, Brown G, Chao C, Chitipiralla S, Gu B, Hart J, Hoffman D, Hoover J (2016). ClinVar: public archive of interpretations of clinically relevant variants. Nucleic Acids Res.

[20] Sabater-Molina M, Guillen-Navarro E, Garcia-Molina E, Ballesta-Martinez MJ, Escudero F, Ruiz-Espejo F (2013). Barth syndrome in adulthood: a clinical case. Rev Esp Cardiol.

[21] Biesecker LG, Green RC (2014). Diagnostic clinical genome and exome sequencing. N Engl J Med.

[22] Miller NA, Farrow EG, Gibson M, Willig LK, Twist G, Yoo B, Marrs T, Corder S, Krivohlavek L, Walter A (2015). A 26-hour system of highly sensitive whole genome sequencing for emergency management of genetic diseases. Genome Med.

[23] Kohler S, Schulz MH, Krawitz P, Bauer S, Dolken S, Ott CE, Mundlos C, Horn D, Mundlos S, Robinson PN (2009). Clinical diagnostics in human genetics with semantic similarity searches in ontologies. Am J Hum Genet.

